# Characterization of Ca^2+^-Dependent SOS-like Pathway Components Involved in the Salt Stress Response of an Intertidal Macroalga, *Pyropia haitanensis*

**DOI:** 10.3390/plants15203188

**Published:** 2026-10-17

**Authors:** Jian Wen, Hongyan Si, Jiaxuan Li, Kai Xu, Yan Xu, Dehua Ji, Jiaying Wang, Wenfei Xian, Chaotian Xie, Wenlei Wang

**Affiliations:** 1 Fisheries College, Jimei University, Xiamen 361021, China; 2 Fujian Engineering Research Center of Aquatic Breeding and Healthy Aquaculture, Xiamen 361021, China; 3 State Key Laboratory of Mariculture Breeding, Fisheries College of Jimei University, Ningde 352100, China

**Keywords:** intertidal macroalgae, *Pyropia haitanensis*, salt stress, Na^+^/H^+^ antiporter, Ca^2+^ signaling, SOS-like pathway

## Abstract

Intertidal red algae show much stronger tolerance to hypersaline stress than terrestrial plants. However, the characteristics of Ca^2+^ signaling and the SOS-related ion regulatory mechanisms involved in their hypersaline stress responses remain unclear. In this study, the representative intertidal red alga *Pyropia haitanensis* was used to characterize Ca^2+^ signal transduction under hypersaline stress and to identify key components of the SOS pathway. Hypersaline stress rapidly induced a Ca^2+^ signal mainly driven by extracellular Ca^2+^ influx, which peaked at 15 min. Inhibition of Ca^2+^ influx significantly suppressed Na^+^ efflux, whereas inhibition of SOS1-mediated Na^+^ efflux enhanced Ca^2+^ influx. These results suggest that SOS1-associated Na^+^ efflux is Ca^2+^-dependent and closely associated with Ca^2+^ signaling during the hypersaline stress response. Based on the reference genome, 14 SOS-related candidate genes were identified. Most of these genes were significantly upregulated at approximately 10 min after hypersaline treatment, showing a rapid response pattern. Among them, *PhSOS1-4* responded significantly at the transcriptional, protein abundance, and phosphorylation levels. PhSOS1-4 interacted with PhSOS3L-5 and PhSOS2L-2, respectively, whereas no direct interaction was detected between PhSOS3L-5 and PhSOS2L-2. These results suggest a possible SOS-like regulatory mode in *P. haitanensis* that is distinct from the classical SOS pathway in higher plants. In this pathway, PhSOS3L-5 may directly interact with PhSOS1-4 and potentially participate in the rapid regulation of Na+ efflux. Heterologous expression showed that both *PhSOS1-4* and *PhSOS3L-5* enhanced the hypersaline tolerance of *Chlamydomonas reinhardtii*. These findings provide new insights into the strong Na^+^ efflux capacity of intertidal red algae.

## 1. Introduction

Soil salinization is a major form of land degradation worldwide. It has affected more than one billion hectares of land and poses a persistent threat to crop yield and agricultural production [1]. In saline soils, plants are usually exposed to sustained salt stress, which can further induce multiple forms of damage, including osmotic stress, ion toxicity, and oxidative stress. Among these effects, ion toxicity caused by excessive Na^+^ accumulation is particularly detrimental [2]. Because Na^+^ and K^+^ share certain physicochemical similarities, excessive Na^+^ entry into cells can interfere with K^+^ uptake and promote K^+^ efflux by altering membrane potential. This disrupts intracellular K^+^/Na^+^ homeostasis and affects normal physiological metabolism [3]. Therefore, limiting cytosolic Na^+^ overaccumulation and maintaining a relatively stable K^+^/Na^+^ ratio are important physiological bases for plant adaptation to salt stress [4].

In plants, the SOS (Salt Overly Sensitive) pathway is an important signaling pathway for maintaining K^+^/Na^+^ homeostasis under salt stress. Salt stress can rapidly alter cytosolic Ca^2+^ levels. This signal is then perceived by the Ca^2+^ sensors SOS3 (Salt Overly Sensitive 3) or SCaBP8 (SOS3-like calcium-binding protein 8), which form a complex with SOS2 (serine/threonine protein kinase). The activated SOS3-SOS2 (or SCaBP8-SOS2) complex further regulates the plasma membrane-localized SOS1, a Na^+^/H^+^ antiporter, thereby promoting the extrusion of excess cytosolic Na^+^, alleviating Na^+^ toxicity, and maintaining cellular K^+^/Na^+^ balance [5]. Hameed et al. (2025) found that CaCl_2_ pretreatment of *Glycine max* seeds before NaCl stress enhanced the expression of *GmSOS*-related genes, accompanied by a reduced Na^+^/K^+^ ratio and improved germination and seedling growth [6]. Exogenous Ca^2+^ treatment also upregulated SOS-related genes in *Lolium perenne* L. under salt stress and improved plant salt tolerance [7]. These studies indicate that the Ca^2+^-mediated SOS pathway plays an important role in plant responses to salt stress.

Unlike most terrestrial plants, intertidal red algae are frequently exposed to rapid salinity fluctuations driven by tidal cycles and show strong tolerance to hypersaline stress. For example, *Pyropia haitanensis* can tolerate extreme hypersaline treatment with approximately 1700 mM NaCl [8]. This tolerance threshold is much higher than the salt stress tolerance levels of terrestrial model plants, such as *Arabidopsis thaliana* [9], rice [10], wheat [11], and maize [12], which are usually exposed to 100–150 mM NaCl. In addition, *P. haitanensis* shows a rapid response to hypersaline stress. Wang et al. [13] combined physiological measurements with transcriptome analysis and found that, under 100‰ hypersaline treatment, *P. haitanensis* achieved stable and rapid osmotic adjustment through the coordinated accumulation of inorganic ions and organic osmolytes, including glycine betaine and trehalose. Notably, trehalose content increased markedly within 15 min after hypersaline treatment and remained at a high level during the subsequent treatment period. In contrast, higher plants often require several hours or even days to synthesize sufficient osmolytes to cope with salt stress [14,15,16,17]. These findings suggest that *P. haitanensis* may have evolved a salt stress response strategy distinct from that of typical terrestrial plants.

Chen et al. [18] used Non-invasive Micro-test Technology (NMT) and pharmacological experiments to demonstrate that hypersaline stress rapidly activates plasma membrane H^+^-ATPase in *P. haitanensis*. This activation enhances the transmembrane proton electrochemical gradient, thereby providing the driving force for Na^+^ efflux mediated by plasma membrane Na^+^/H^+^ antiporters. Meanwhile, H^+^-ATPase-mediated plasma membrane hyperpolarization alleviates salt-induced membrane depolarization, thereby reducing K^+^ efflux mediated by depolarization-activated outward-rectifying K^+^ channels and ultimately contributing to the maintenance of a higher intracellular K^+^/Na^+^ ratio. These findings indicate that SOS1-mediated Na^+^ efflux and K^+^/Na^+^ homeostasis are important components of salt stress adaptation in *P. haitanensis*. However, compared with the classical Ca^2+^-activated SOS3-SOS2-SOS1 cascade in terrestrial plants, the Ca^2+^-dependent regulation of SOS1-mediated Na^+^ efflux in *P. haitanensis* and whether SOS candidate proteins form a similar regulatory cascade remain unclear. Therefore, this study focused on Ca^2+^ signaling and the SOS pathway during hypersaline stress responses in *P. haitanensis*. By integrating NMT, genome-wide identification, gene cloning, yeast two-hybrid assays, and heterologous expression, we characterized hypersaline-induced Ca^2+^ signaling dynamics and identified key SOS components involved in salt stress responses and their interaction networks. These findings provide new insights into hypersaline adaptation and SOS-related ion homeostasis regulation in intertidal macroalgae.

## 2. Materials and Methods

### 2.1. Experimental Materials and Treatments

Thalli of *P. haitanensis* strain W28 were obtained from the *P. haitanensis* Germplasm Resource Bank in Fujian Province [19]. Thalli with a length of 15 ± 3 cm, intact morphology, and consistent growth status were selected for subsequent experiments. The thalli were cultured in 30‰ natural seawater under the following conditions: 21 ± 1 °C, a light intensity of 50–60 μmol/(m^2^·s), and a photoperiod of 12 h light/12 h dark. The culture medium was replaced every 2 d, and the thalli were continuously aerated during cultivation. Artificial seawater was prepared according to the method described by Zheng et al. (2022) [20]. Ca^2+^-free artificial seawater was prepared with the same components except that CaCl_2_ was omitted.

Hypersaline stress treatment was performed using 110‰ artificial seawater. Thalli with consistent growth status were placed in 1 L conical flasks, with six thalli in each flask. The treatment times were set as 0, 5, 10, 15, 30, 60, 120, and 240 min. For thalli under Ca^2+^-free conditions, Ca^2+^ was supplemented after 30 min, 1 h, 2 h, and 4 h of hypersaline stress to a final concentration of 10 mM. Pharmacological treatments were performed using 10 μM Verapamil, a Ca^2+^ channel inhibitor, and 5 μM 2-APB, an inhibitor of intracellular Ca^2+^ store release, to analyze the source of Ca^2+^ and its role in the hypersaline stress response.

The cell wall-deficient *C. reinhardtii* strain CC-400 cw15 mt^+^ was used for heterologous expression experiments. *C. reinhardtii* was cultured in TAP (Tris-acetate-phosphate) liquid medium under the following conditions: 25 °C, 70 rpm, a light intensity of 50 μmol/(m^2^·s), and a photoperiod of 14 h light/10 h dark. When the cells reached the logarithmic growth phase, corresponding to an OD_750_ of approximately 0.5, they were used for subsequent transformation and salt stress experiments [21].

### 2.2. Measurement of the Chlorophyll Fluorescence Parameter Fv/Fm

The chlorophyll fluorescence parameter of PSII was measured using a Diving-PAM fluorometer (Walz, Effeltrich, Germany). Before measurement, the thalli were dark-adapted for 10 min. The thalli were then fixed at approximately one-third of their length using a leaf clip, and measurements were performed after the instrument parameters were adjusted. The initial fluorescence (F_0_) and maximum fluorescence (Fm) were measured to calculate variable fluorescence (Fv = Fm − F_0_), and the maximum photochemical efficiency of PSII was then calculated as *Fv/Fm*. Four independent biological replicates were included for each treatment.

### 2.3. Measurement of Ca^2+^, K^+^, and Na^+^ Ion Fluxes

To analyze the effects of salt stress and inhibitor treatments on ion transport in thalli, Ca^2+^, K^+^, and Na^+^ fluxes were measured using Non-invasive Micro-test Technology (NMT; YoungerUSA LLC, Amherst, MA, USA). The preparation of the measuring solutions and the ion flux detection procedure were performed according to previous NMT studies in *P. haitanensis* [18,22]. During the experiment, healthy thalli of *P. haitanensis* with a length of 15 ± 3 cm were placed in Petri dishes containing an appropriate volume of equilibrium solution and fixed to the bottom of the dishes with agar blocks. After equilibration for approximately 1 min, ion fluxes were continuously measured for 6 min using the corresponding ion-selective microelectrodes. The results were presented as the mean flux values measured at the same time point from thalli under the same treatment condition. Six independent biological replicates were included for each treatment.

### 2.4. Genome-Wide Identification of SOS Candidate Genes in P. haitanensis

To identify candidate genes related to the SOS pathway in *P. haitanensis*, the SOS1, SOS2, and SOS3 protein sequences from *Arabidopsis thaliana*, rice, tomato, and potato were first downloaded from the NCBI database. These sequences included AtSOS1 (NP_178307.2), AtSOS2 (NP_198391.1), and AtSOS3 (NP_001331303.1) from A. thaliana; OsSOS1 (AWX66759.1), OsSOS2 (ACD76985.1), and OsSOS3 (ABB52102.1) from rice; SlSOS1 (NP_001234698.2), SlSOS2 (NP_001234210.1), and SlSOS3 (NP_001234705.1) from tomato; and StSOS1 (XP_006364070.1), StSOS2 (XP_006345226.1), and StSOS3 (XP_006350549.1) from potato. Using these sequences as queries, BLASTP was performed against the predicted protein sequences of *P. haitanensis* to preliminarily screen SOS-related candidate proteins.

Meanwhile, hidden Markov models of characteristic domains related to SOS1 (PF00999), SOS2 (PF03822 and PF00069), and SOS3 (PF13499) were downloaded from the Pfam database, and HMMER 3.0 was used to search these domains against the predicted protein sequences of *P. haitanensis*. The screening threshold was set to E-value < 1 × 10^−5^. The candidate proteins were then integrated and filtered based on the BLASTP homology results, HMMER domain search results, and gene functional annotation information. Finally, the conserved domains of the candidate proteins were verified using NCBI CD-search. Proteins supported by homologous alignment, consistent functional annotation, and the corresponding target domains were retained and identified as SOS pathway-related candidate gene members in *P. haitanensis*. The subcellular localization of the identified SOS candidate proteins was predicted using WoLF PSORT (https://wolfpsort.hgc.jp/) with default parameters.

### 2.5. Analysis of Phosphorylation Modification Information of Sos Candidate Proteins

The phosphorylation modification information of SOS candidate proteins was obtained from previously generated phosphoproteomic data of *P. haitanensis* under hypersaline stress in our laboratory [23]. In this study, differentially phosphorylated sites and relative phosphorylation levels were extracted according to the IDs of PhSOS candidate proteins and used for subsequent visualization analysis.

### 2.6. Total Rna Extraction and Cdna Synthesis

Thalli of *P. haitanensis* or cells of *C. reinhardtii* were ground into powder in liquid nitrogen. Total RNA was extracted using a Plant RNA Extraction Kit (Omega Bio-tek, Norcross, GA, USA). RNA integrity was examined by 1% agarose gel electrophoresis, and RNA purity and concentration were assessed by measuring the OD_260_/OD_280_ ratio. The RNA was then reverse-transcribed into cDNA using the Evo M-MLV RT Mix Kit (Accurate Biology, Changsha, China). The resulting cDNA was diluted 10-fold and stored for subsequent qRT-PCR analysis.

### 2.7. Quantitative Real-Time Pcr

To analyze the expression changes in SOS candidate genes in *P. haitanensis* under salt stress and inhibitor treatments, gene-specific qPCR primers were designed based on the gene sequences, with *PhUBC* used as the internal reference gene. The primers used in this study are listed in Table S1. The qPCR reaction system had a total volume of 20 μL, including 10 μL of 2× SYBR Green Pro Taq HS Premix, 0.4 μL of forward primer, 0.4 μL of reverse primer, 2 μL of diluted cDNA template, and 7.2 μL of ddH_2_O. The amplification program was as follows: pre-denaturation at 95 °C for 30 s, followed by 40 cycles of 95 °C for 5 s and 60 °C for 30 s. After amplification, melting curve analysis was performed to confirm amplification specificity [21]. Three independent biological replicates were included for each treatment.

### 2.8. Western Blot Analysis

*P. haitanensis* thalli were thoroughly ground on ice. NP40 lysis buffer (N8030-100, Solarbio, Beijing, China) was added at a ratio of 30–50 μL per 5 mg of thallus tissue, followed by the addition of protease inhibitor and phosphatase inhibitor at ratios of 1:50 and 1:100, respectively. The samples were lysed for 15 min. The lysates were centrifuged at 12,000× *g* for 15 min at 4 °C, and the supernatants were collected. Protein concentration was determined using a BCA Protein Assay Kit (PC0020, Solarbio, Beijing, China). Equal amounts of protein samples were mixed with 5× loading buffer, denatured at 100 °C for 5–10 min, and centrifuged. The supernatants were used for SDS-PAGE, with 30 μg of protein loaded per lane.

After electrophoresis, proteins were transferred onto PVDF membranes at 80 V for 1 h. The membranes were washed with TBST and blocked with 5% BSA at room temperature for 1 h. The corresponding primary antibodies were then added, and the membranes were incubated overnight at 4 °C. After washing with TBST, the membranes were incubated with Goat anti-Rabbit IgG secondary antibody (Solarbio, Beijing, China) at room temperature for 1 h. Rubisco large subunit, form I (RbcL; Agrisera, Vännäs, Sweden; AS03037), was used as the loading control. Finally, signals were detected using an ECL chemiluminescence detection kit (E411-04, Vazyme, Nanjing, China) and captured using an imaging system.

### 2.9. Yeast Two-Hybrid Validation

PhSOS1-4 and its truncated fragments were cloned into the pTSU2-APP vector as bait proteins, whereas candidate interacting proteins or *P. haitanensis* cDNA library fragments were cloned into the pPR3-N vector as prey proteins. Yeast transformation, selective culture, and colorimetric detection were performed according to a previously reported yeast two-hybrid screening system [21]. The combination of pTSU2-APP Control Vector and pPR3-N Control Vector was used as the negative control, and the combination of pTSU2-APP Control Vector and pNubG-Fe65 Control Vector was used as the positive control for autoactivation and toxicity tests of the bait protein.

During library screening, the transformation products were plated on selective medium containing X-Gal and incubated at 30 °C for 3–5 d. Positive clones showing blue coloration were selected. Candidate interacting proteins were identified after plasmid recovery, amplification, sequencing, and sequence alignment. Subsequently, the candidate prey plasmids and the corresponding bait plasmids were co-transformed into yeast again and subjected to one-to-one interaction validation on QDO/X/A selective medium. Clones that grew normally and showed blue coloration were considered positive interactions.

### 2.10. Heterologous Expression in C. reinhardtii and Salt Tolerance Assay

Because a stable genetic transformation system for *Pyropia* has not yet been well established, *C. reinhardtii* was used as a heterologous expression system to validate the salt tolerance function of key SOS components from *P. haitanensis* [24,25]. The CDS sequences of *PhSOS1-4*, *PhSOS2L-2*, and *PhSOS3L-5* were separately cloned into the pChlamy_3 expression vector. KpnI/PstI sites were used for *PhSOS2L-2* and *PhSOS3L-5*, whereas KpnI/NdeI sites were used for *PhSOS1-4*.

After verification by colony PCR and sequencing, the recombinant plasmids were introduced into wild-type *C. reinhardtii* using the glass bead transformation method. Positive transformants were screened on TAP medium containing hygromycin, and the transformation and screening procedures were performed according to previously reported methods [25]. Positive transformants verified by RT-PCR were used for subsequent functional analysis. For salt tolerance phenotypic evaluation, wild-type and transgenic *C. reinhardtii* were cultured under 225 mM NaCl stress, and growth phenotypes were observed at 0, 24, 48, 72, and 96 h. OD_750_ was measured to evaluate changes in biomass. Three independent biological replicates were used for OD_750_ measurements for each treatment. For target gene expression analysis, samples were collected at 0, 15, and 30 min after 225 mM NaCl treatment, and the expression levels of *PhSOS1-4*, *PhSOS2L-2*, and *PhSOS3L-5* were analyzed by qRT-PCR using β-tubulin as the internal reference gene. Three independent biological replicates were included for each qRT-PCR analysis.

### 2.11. Statistical Analysis

All statistical analyses were performed using IBM SPSS Statistics software (version 26.0; IBM Corp., Armonk, NY, USA). Quantitative data are presented as the mean ± standard deviation (SD). Differences among multiple groups were evaluated using one-way analysis of variance (ANOVA), followed by Fisher’s least significant difference (LSD) test for post-hoc multiple comparisons. Differences were considered statistically significant at *p* < 0.05 and highly significant at *p* < 0.01.

## 3. Results

### 3.1. Effects of Ca^2+^ Availability on the Early Physiological Response of P. haitanensis Under Hypersaline Stress

**Figure 1:**
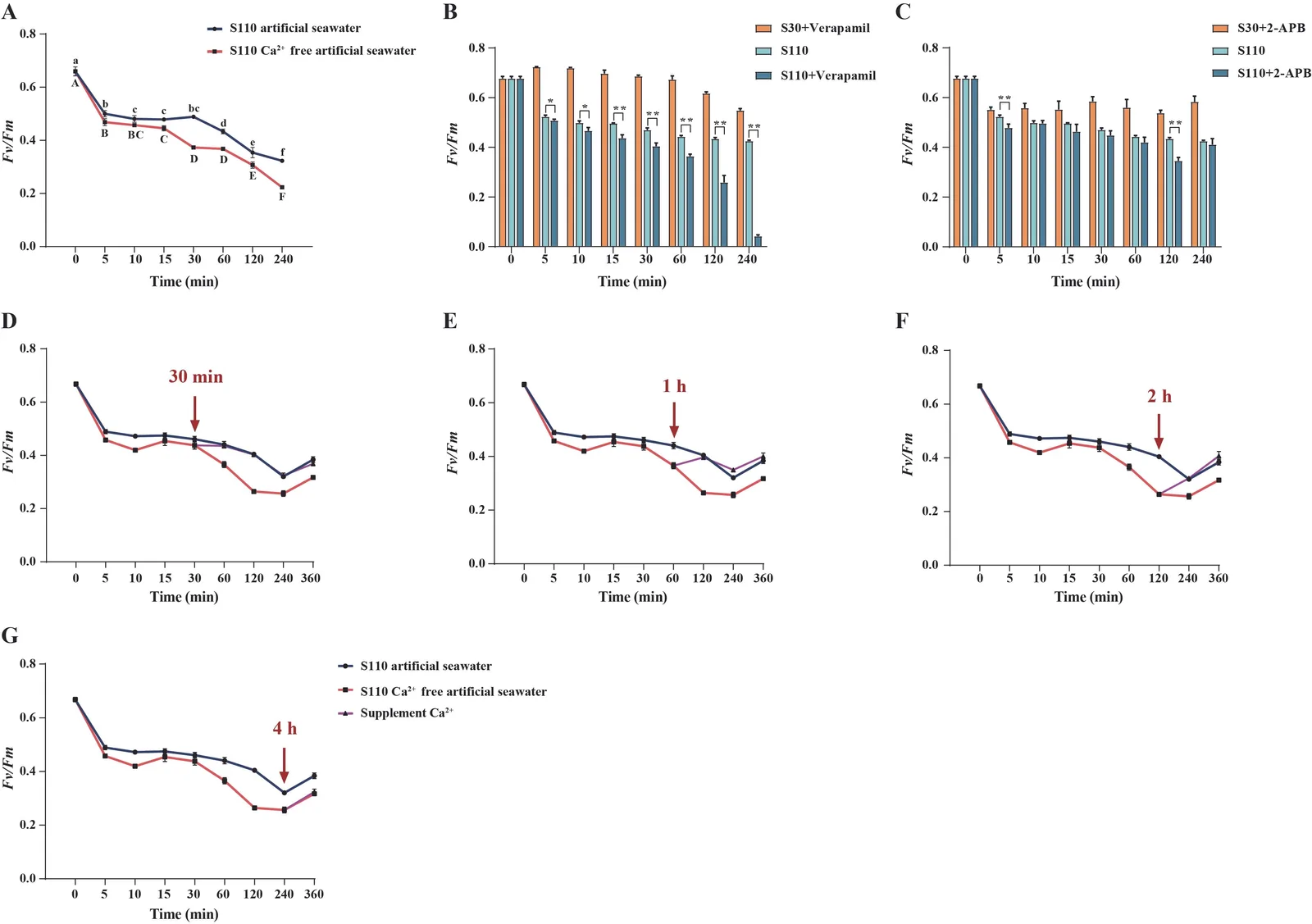
Effects of Ca^2+^ availability on the early physiological response of *P. haitanensis* under hypersaline stress. (**A**) Changes in *Fv/Fm* of *P. haitanensis* under hypersaline artificial seawater with or without Ca^2+^. (**B**) Effects of the plasma membrane Ca^2+^ channel inhibitor Verapamil on *Fv/Fm* under hypersaline stress. (**C**) Effects of the intracellular Ca^2+^ store release inhibitor 2-APB on *Fv/Fm* under hypersaline stress. (**D**–**G**): Changes in *Fv/Fm* after Ca^2+^ supplementation at 30 min, 1 h, 2 h, and 4 h under Ca^2+^-free hypersaline stress. S30 indicates treatment with 30‰ normal seawater; S110 indicates 110‰ hypersaline treatment; Ca^2+^ free indicates Ca^2+^-free treatment; supplement Ca^2+^ indicates Ca^2+^ supplementation. Different letters indicate significant differences among different time points within the same treatment (*p* < 0.05). Asterisks indicate significant differences between treatments, with * and ** indicating *p* < 0.05 and *p* < 0.01, respectively.

To evaluate the effects of Ca^2+^ availability on the early physiological response of *P. haitanensis* under hypersaline stress, thalli were treated with 110‰ artificial hypersaline seawater with Ca^2+^ (S110 artificial seawater) or without Ca^2+^ (S110 Ca^2+^ free artificial seawater) for 0, 5, 10, 15, 30, 60, 120, and 240 min. Under the Ca^2+^-containing hypersaline treatment, *Fv/Fm* remained relatively stable within the first 30 min and then decreased significantly with prolonged stress. In contrast, under the Ca^2+^-free hypersaline treatment, *Fv/Fm* remained relatively stable only within the first 15 min and decreased significantly thereafter (Figure 1A).

Meanwhile, thalli were treated with Verapamil, a plasma membrane Ca^2+^ channel inhibitor, and 2-APB, an antagonist of intracellular Ca^2+^ store release, to distinguish the relative roles of extracellular Ca^2+^ influx and intracellular Ca^2+^ store release in the hypersaline stress response. The results showed that, after Verapamil was added under hypersaline treatment (S110 + Verapamil), *Fv/Fm* gradually decreased with prolonged stress and reached 0.04 at 240 min. In contrast, after 2-APB treatment (S110 + 2-APB), *Fv/Fm* decreased significantly only at 5 min and 2 h, with no significant changes at the other time points. These results suggest that extracellular Ca^2+^ availability plays a more prominent role than intracellular Ca^2+^ release in maintaining the physiological status of *P. haitanensis* under hypersaline stress (Figure 1B,C).

To further evaluate the time window during which Ca^2+^ availability contributes to the maintenance and recovery of physiological status under hypersaline stress, Ca^2+^ was supplemented to the standard concentration of artificial seawater after 30 min, 1 h, 2 h, and 4 h of Ca^2+^-free hypersaline stress. The results showed that Ca^2+^ supplementation after 30 min, 1 h, and 2 h restored *Fv/Fm* to levels similar to those of the hypersaline treatment with Ca^2+^ (Figure 1D–F). However, Ca^2+^ supplementation after 4 h of stress did not restore *Fv/Fm*, which remained at a low level (Figure 1G).

### 3.2. Dynamic Changes in Ca^2+^ Signaling and Its Association with Na^+^ and K^+^ Fluxes

**Figure 2:**
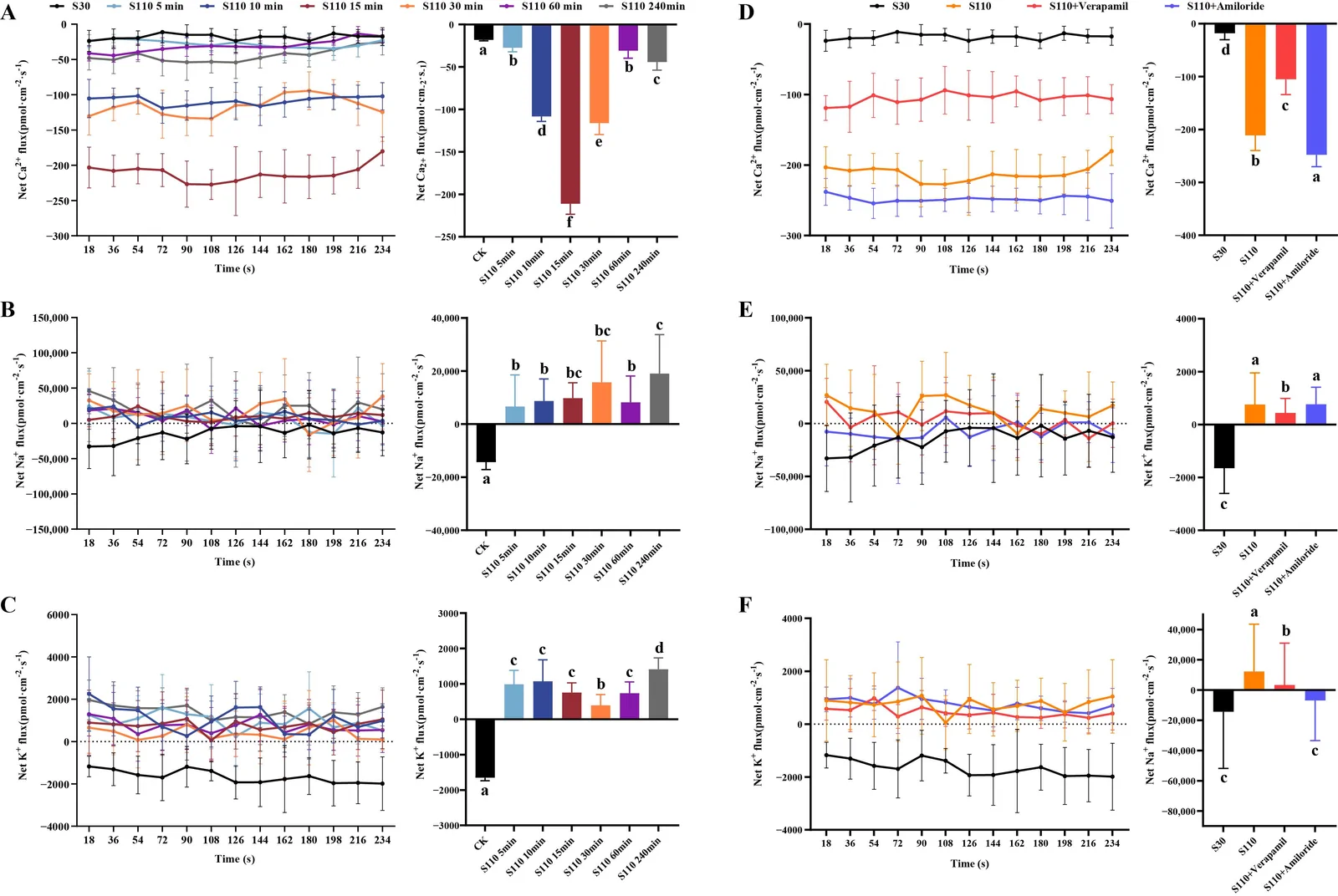
Characteristics of Ca^2+^ influx and Na^+^ and K^+^ flux changes in *P. haitanensis* under hypersaline stress. (**A**) Dynamic changes in Ca^2+^ flux in *P. haitanensis* under normal seawater and different durations of hypersaline treatment. (**B**) Dynamic changes in Na^+^ flux in *P. haitanensis* under normal seawater and different durations of hypersaline treatment. (**C**) Dynamic changes in K^+^ flux in *P. haitanensis* under normal seawater and different durations of hypersaline treatment. (**D**–**F**) Effects of Verapamil and Amiloride on Ca^2+^, Na^+^, and K^+^ fluxes after 15 min of hypersaline stress. The left side of each panel shows the dynamic changes in ion flux during continuous monitoring, and the right side shows the mean ion flux under the corresponding treatment. S30 indicates treatment with 30‰ normal seawater; S110 indicates 110‰ hypersaline treatment; S110 + Verapamil indicates hypersaline treatment with the plasma membrane Ca^2+^ channel inhibitor Verapamil; S110 + Amiloride indicates hypersaline treatment with the SOS1-related Na^+^/H^+^ antiporter inhibitor Amiloride. Positive values indicate ion efflux, and negative values indicate ion influx. Different letters indicate significant differences among treatments (*p* < 0.05).

To analyze the dynamic characteristics of Ca^2+^ signaling in *P. haitanensis* during hypersaline stress responses, Ca^2+^ flux was monitored in real time using NMT. The results showed that, compared with the normal treatment (S30), hypersaline stress (S110) significantly induced Ca^2+^ influx. The Ca^2+^ influx peaked at 15 min and then gradually decreased, but remained significantly higher than the control level (Figure 2A). Meanwhile, Na^+^ and K^+^ both showed influx under normal conditions, whereas both shifted to efflux under hypersaline stress (Figure 2B,C).

Subsequently, Verapamil and the SOS1-related Na^+^/H^+^ antiporter inhibitor Amiloride were added under 110‰ hypersaline treatment, and ion fluxes were measured after 15 min to examine the relationship between Ca^2+^ signaling and Na^+^/K^+^ fluxes. The results showed that, compared with the normal hypersaline treatment (S110), Verapamil significantly inhibited Ca^2+^ influx and also significantly suppressed K^+^ and Na^+^ efflux (Figure 2D–F). In contrast, Amiloride treatment (S110 + Amiloride) significantly enhanced Ca^2+^ influx. K^+^ flux showed no significant change, whereas Na^+^ flux shifted from efflux to influx (Figure 2D–F).

### 3.3. Identification and Expression Pattern Analysis of Sos Pathway-Related Genes

**Figure 3:**
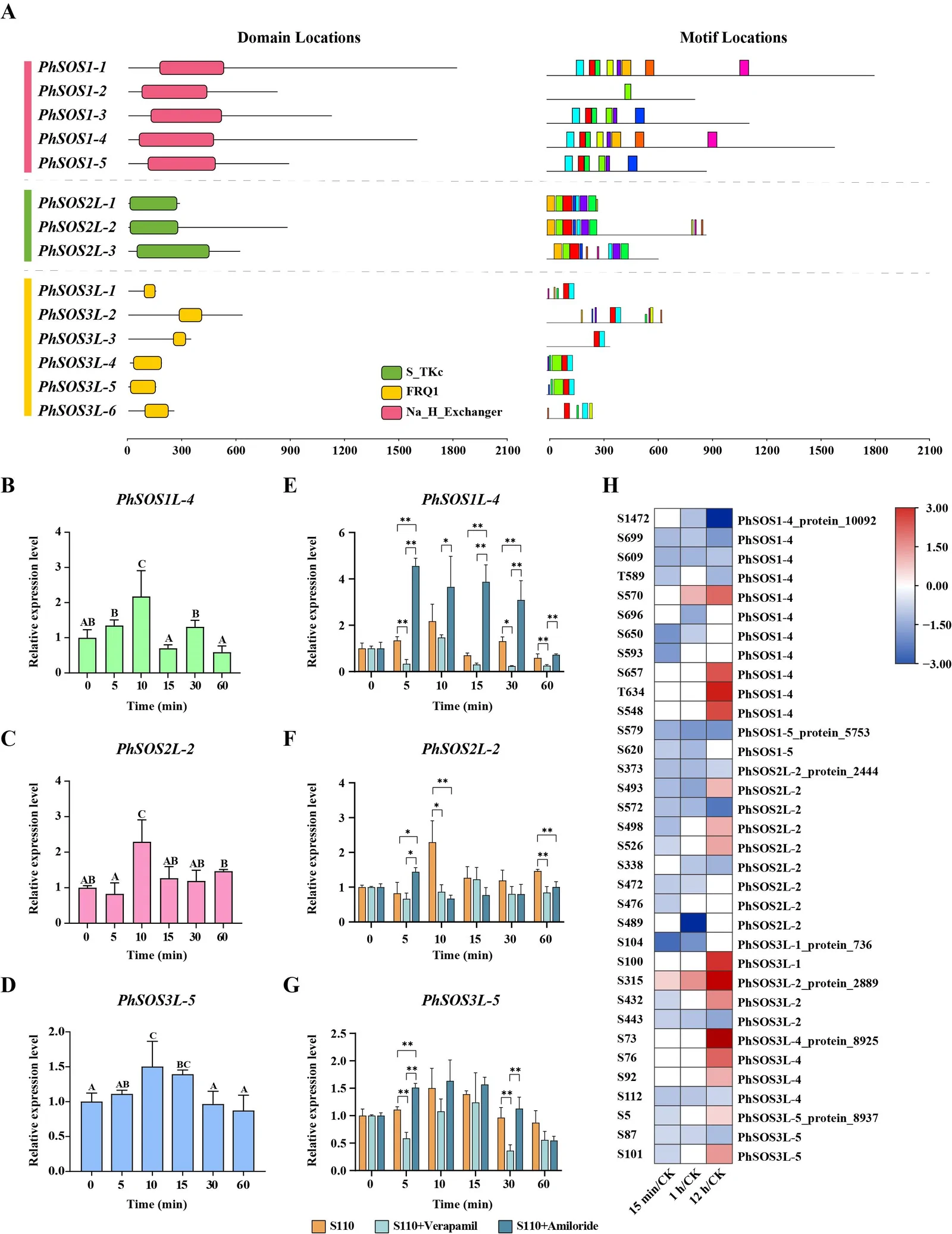
Structural features and hypersaline response patterns of SOS candidate components in *P. haitanensis*. (**A**) Conserved domains and motif distributions of PhSOS1, PhSOS2L, and PhSOS3L candidate proteins in *P. haitanensis*. (**B**–**D**) Expression changes in the representative SOS candidate genes *PhSOS1-4*, *PhSOS2L-2*, and *PhSOS3L-5* under hypersaline stress. (**E**–**G**) Expression patterns of *PhSOS1-4*, *PhSOS2L-2*, and *PhSOS3L-5* under hypersaline stress after Verapamil and Amiloride treatments. (**H**) Differential phosphorylation patterns of SOS candidate proteins in *P. haitanensis* under hypersaline stress. Different letters indicate significant differences among different treatment times (*p* < 0.05). * and ** indicate significant and highly significant differences between treatments, respectively (*p* < 0.05 and *p* < 0.01).

Based on the reference genome of *P. haitanensis*, a total of 14 SOS pathway-related candidate genes were identified, including 5 *PhSOS1*, 3 *PhSOS2*, and 6 *PhSOS3* candidate genes. All 5 PhSOS1 proteins contained the Na_H_Exchanger domain, a typical structural feature of previously reported SOS1 proteins (Figure 3A). All 3 PhSOS2 proteins contained a serine/threonine protein kinase domain (S_TKc); however, the C-terminal CIPK_C regulatory domain characteristic of typical CIPK/SOS2 proteins was not detected (Figure 3A). Based on the conserved protein kinase domain and the absence of the canonical CIPK_C domain, these candidates were therefore classified as PhSOS2-like (PhSOS2L) proteins. All 6 PhSOS3 proteins contained the FRQ1 domain, which is associated with Ca^2+^ binding and belongs to the EF-hand superfamily, and their core domain features were consistent with those of SOS3 calcium-sensing proteins (Figure 3A). However, further comparison of EF-hand motif numbers showed that only PhSOS3-4 and PhSOS3-5 contained 4 EF-hand motifs, whereas the remaining members contained only 2–3 EF-hand motifs. This pattern differed from that of typical SOS3 proteins, which usually contain 4 EF-hand motifs (Figure S1). Accordingly, based on their overall similarity to SOS3 proteins and the observed variation in EF-hand motif number, these candidates were classified as PhSOS3-like (PhSOS3L) proteins.

To further annotate the identified SOS candidate proteins, their subcellular localization was predicted using WoLF PSORT. All five PhSOS1 proteins were predominantly predicted to localize to the plasma membrane. In contrast, PhSOS2L and PhSOS3L candidate proteins showed more diverse predicted localization patterns, including the chloroplast, cytoplasm, nucleus, and mitochondria (Table S2).

Quantitative analysis of candidate gene expression under hypersaline stress showed that *PhSOS1-2* was specifically expressed in the filamentous generation (Figure S2), whereas *PhSOS2L-3* and *PhSOS3L-3* showed extremely low expression levels (Ct > 35). These genes were therefore excluded from subsequent analysis. Among the remaining candidate genes, all except *PhSOS2L-1* showed a significant upregulation trend during the early stage of hypersaline stress, approximately at 10 min (Figure 3B–D and Figure S3). Expression analysis after inhibitor treatments showed that the four *PhSOS1* candidate genes were downregulated at all time points under Verapamil treatment, whereas Amiloride treatment significantly inhibited only the expression of *PhSOS1-3* (Figure 3E and Figure S4). Among the *PhSOS2L* genes, *PhSOS2L-1* was not induced by salt stress but was significantly upregulated under Amiloride treatment. *PhSOS2L-2* was induced by salt stress and was significantly suppressed at 10 and 60 min under both Verapamil and Amiloride treatments (Figure 3F and Figure S4). In addition, among the five *PhSOS3L* candidate genes, all genes except *PhSOS3L-6* showed different degrees of downregulation under Verapamil treatment, whereas they generally showed an upregulated trend under Amiloride treatment (Figure 3G and Figure S4).

Based on previously obtained phosphoproteomic data of *P. haitanensis* under hypersaline stress in our laboratory, the phosphorylation modification information of SOS candidate proteins was further analyzed. The results showed that, among PhSOS1 proteins, only PhSOS1-4 and PhSOS1-5 underwent differential phosphorylation. Among PhSOS2L proteins, only PhSOS2L-2 was phosphorylated. Among PhSOS3L proteins, PhSOS3L-1, PhSOS3L-2, PhSOS3L-4, and PhSOS3L-5 showed differential phosphorylation (Figure 3H). Notably, PhSOS1-4 not only contained the largest number of differential phosphorylation sites, with 11 sites identified, but was also significantly induced by salt stress at both the transcript and protein levels (Figure 3E and Figure S5). Therefore, PhSOS1-4 was selected as the core entry point for further analysis of the interaction network of the SOS pathway in *P. haitanensis*.

### 3.4. Identification of Phsos1-4-Interacting Proteins

**Figure 4:**
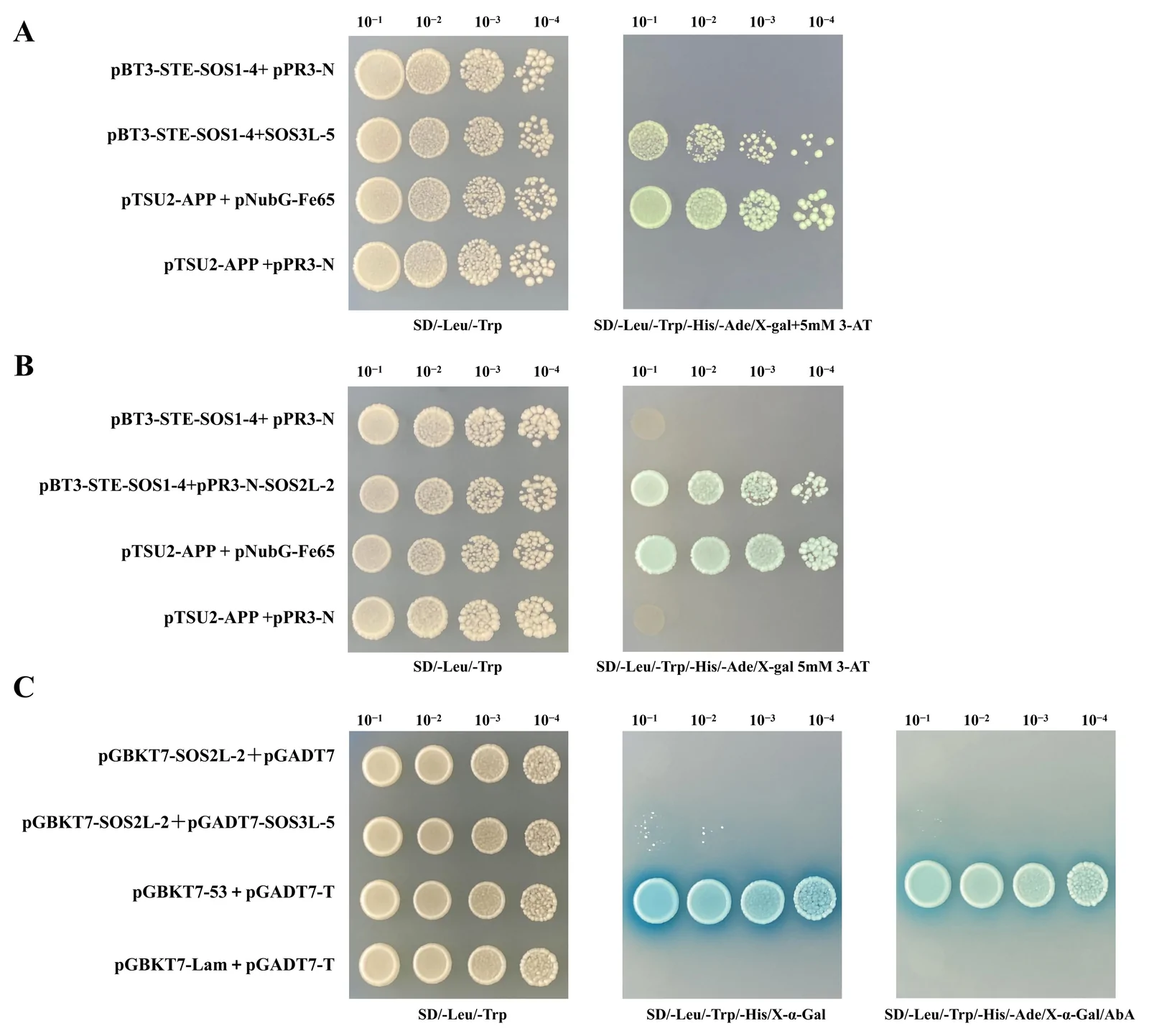
Yeast two-hybrid analysis of PhSOS1-4-centered interactions among SOS candidate proteins. (**A**) Validation of the interaction between PhSOS1-4 and PhSOS3L-5. (**B**) Validation of the interaction between PhSOS1-4 and PhSOS2L-2. (**C**) Detection of the interaction between PhSOS2L-2 and PhSOS3L-5. SD/-Leu/-Trp medium was used to confirm plasmid co-transformation, and high-stringency selective medium containing His, Ade, and the chromogenic substrate was used to assess protein–protein interactions.

Using PhSOS1-4 as the bait protein, a yeast two-hybrid cDNA library screen was performed. A total of 105 primary clones were obtained, of which 99 candidate positive clones showed growth and blue coloration on the selective medium. After sequencing, 50 valid clones were identified (Table S3). Among them, one clone was identified as PhSOS3L-5, and its interaction with PhSOS1-4 was further confirmed by one-to-one Y2H retransformation (Figure 4A).

To further analyze the structural basis of this interaction, PhSOS1-4 was subjected to truncation analysis. The results showed that the N-terminal 1-54 aa fragment of PhSOS1-4 interacted with PhSOS3L-5, whereas the 55-1593 aa region containing the major domains showed no interaction. This indicates that the interaction mainly depends on the N-terminal non-domain region of PhSOS1-4 (Figure S6). In addition, targeted interaction assays showed that PhSOS1-4 also interacted stably with PhSOS2L-2, and this result was further supported by retransformation validation (Figure 4B). In contrast, no direct interaction was detected between PhSOS2L-2 and PhSOS3L-5 (Figure 4C).

### 3.5. Heterologous Expression Analysis of Key Sos-Related Components Under Salt Stress

**Figure 5:**
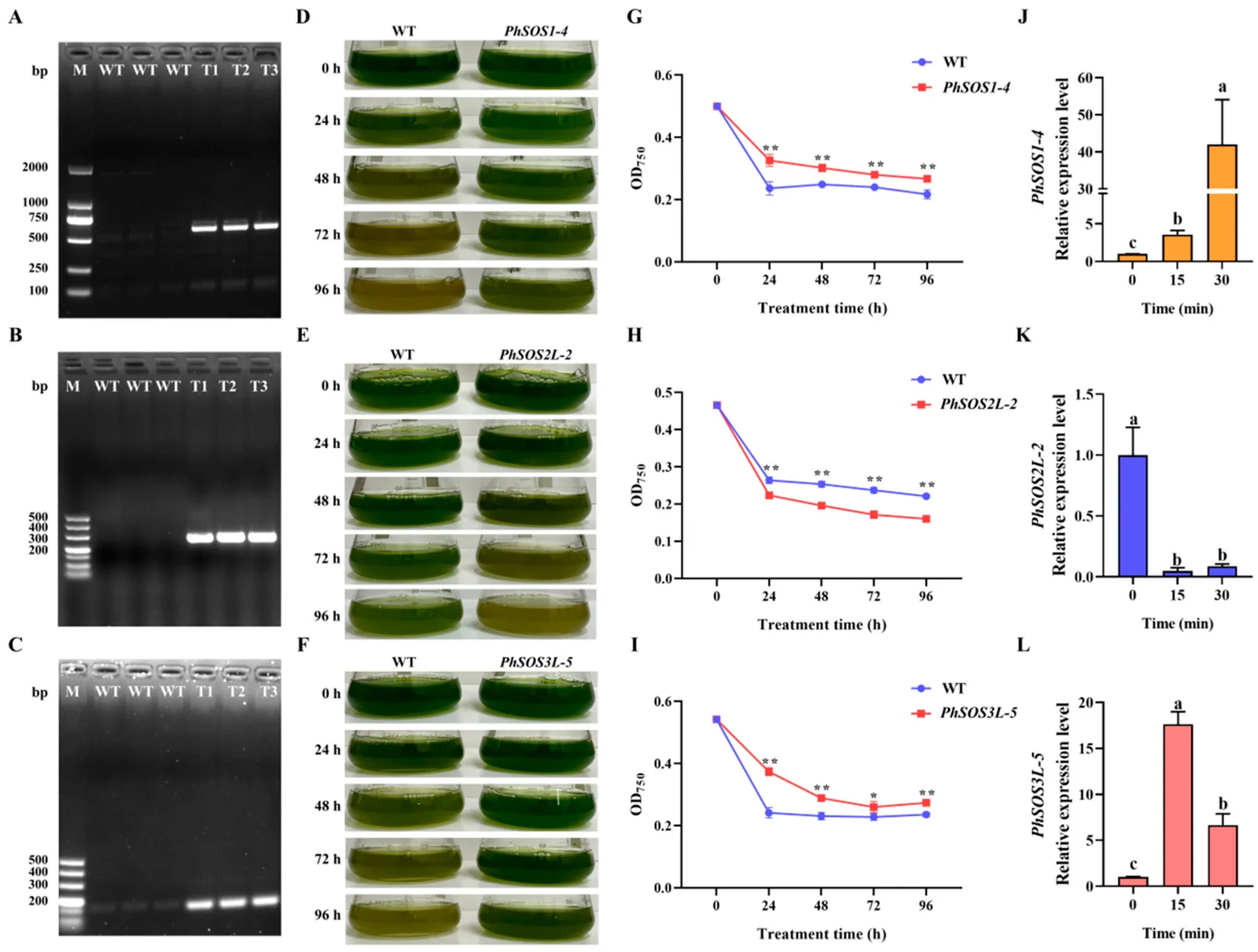
Heterologous expression analysis of key SOS genes from *P. haitanensis* under salt stress. (**A**–**C**) PCR verification of target genes in transgenic *C. reinhardtii*, corresponding to *PhSOS1-4*, *PhSOS2L-2*, and *PhSOS3L-5*, respectively; (**D**–**F**) Growth phenotypes of wild-type and transgenic *C. reinhardtii* under 225 mM NaCl treatment, corresponding to *PhSOS1-4-*, *PhSOS2L-2-*, and *PhSOS3L-5*-transgenic lines, respectively. (**G**–**I**) OD_750_ changes in wild-type and transgenic *C. reinhardtii* at 0, 24, 48, 72, and 96 h after high-salt treatment. (**J**–**L**) Relative expression levels of exogenous *PhSOS1-4*, *PhSOS2L-2*, and *PhSOS3L-5* in transgenic *C. reinhardtii* at 0, 15, and 30 min after high-salt treatment. WT indicates wild-type *C. reinhardtii*; T1–T3 indicate independent transgenic lines; M indicates DNA marker. * and ** indicate significant (*p* < 0.05) and highly significant (*p* < 0.01) differences between transgenic lines and wild type, respectively; different letters indicate significant differences among different treatment time points (*p* < 0.05).

After the *PhSOS1-4*-associated candidate interactions were identified, the three SOS-related genes *PhSOS1-4*, *PhSOS2L-2*, and *PhSOS3L-5* were individually introduced into *Chlamydomonas reinhardtii* for heterologous expression analysis to further evaluate their roles in the salt stress response of *P. haitanensis.* The results showed that positive fragments corresponding to the target genes were amplified in all three types of transgenic *C. reinhardtii*, whereas no corresponding bands were detected in the wild type. This indicated that the target genes were successfully introduced into *C. reinhardtii*. Representative T1 transformants were then selected for subsequent analysis (Figure 5A–C).

After high-salt treatment with 225 mM NaCl, the growth phenotypes of the transgenic lines showed significant changes compared with the wild type. Specifically, *PhSOS1-4-* and *PhSOS3L-5*-transgenic *C. reinhardtii* maintained better growth at all time points (Figure 5D,F), and their OD_750_ values were significantly higher than those of the wild type (Figure 5G,I). These results support a positive contribution of *PhSOS1-4* and *PhSOS3L-5* to salt tolerance in *C. reinhardtii*. In contrast, the growth of *PhSOS2L-2*-transgenic *C. reinhardtii* was significantly inhibited after high-salt treatment, and its OD_750_ value was significantly lower than that of the wild type (Figure 5E,H). Consistent with these phenotypic changes, *PhSOS3L-5* and *PhSOS1-4* were both significantly induced after salt stress. *PhSOS3L-5* reached its expression peak at 15 min, whereas *PhSOS1-4* reached its highest expression level at 30 min, with a relative expression level approximately 42-fold higher than that in the control group (Figure 5J,L). By contrast, *PhSOS2L-2* showed a significant downregulation trend after salt stress (Figure 5K).

## 4. Discussion

### 4.1. Early Response Characteristics of Ca^2+^ Signaling in P. haitanensis Under Hypersaline Stress

Ca^2+^ is an important intracellular second messenger that can undergo rapid dynamic changes in response to external stimuli and participate in plant perception and responses to environmental stress by regulating ion channels, protein kinases, and stress-related gene expression [26]. *Fv/Fm* reflects the maximum photochemical efficiency of PSII reaction centers and is widely used in plant stress studies to evaluate photosystem damage and the degree of physiological injury [27,28]. In this study, *Fv/Fm* in *P. haitanensis* under hypersaline treatment with Ca^2+^ was generally higher than that under Ca^2+^-free treatment, and the decline in *Fv/Fm* was significantly delayed (Figure 1A). This indicates that the presence of Ca^2+^ helps alleviate early physiological damage caused by hypersaline stress. This result is consistent with reports in terrestrial plants such as rice and wheat, in which exogenous Ca^2+^ alleviates salt stress-induced damage [29,30], supporting an important role of Ca^2+^ availability in maintaining physiological performance during the early hypersaline stress response in *P. haitanensis*.

Different stress stimuli can induce Ca^2+^ signals with distinct temporal, spatial, and amplitude characteristics, and this dynamic specificity is an important basis for Ca^2+^ to function as a second messenger in distinguishing external stimuli and activating corresponding response processes [31,32]. Zheng et al. [22] found that heat stress induced Ca^2+^ influx in *P. haitanensis*, which peaked at 15 min at approximately 38 pmol·cm^−2^·s^−1^ and then returned to the normal physiological level after 30 min. In comparison, the present study found that hypersaline stress also induced Ca^2+^ influx and reached a peak at 15 min, but the peak intensity was approximately 211 pmol·cm^−2^·s^−1^ and remained significantly higher than the normal treatment level throughout the subsequent treatment period until 240 min (Figure 2A). These results indicate that Ca^2+^ signaling in *P. haitanensis* is rapidly activated under both heat and hypersaline stress. However, hypersaline stress-induced Ca^2+^ influx showed a higher amplitude and stronger persistence, suggesting that *P. haitanensis* may distinguish different types of stress stimuli through differential Ca^2+^ signaling dynamics and thereby initiate corresponding downstream response processes.

In plant cells, increased cytosolic Ca^2+^ mainly originates from extracellular Ca^2+^ influx through plasma membrane Ca^2+^ channels or from Ca^2+^ release from intracellular stores such as the vacuole [33,34]. Pharmacological analysis showed that Verapamil treatment significantly aggravated the decline in *Fv/Fm* under hypersaline stress, whereas 2-APB treatment caused only stage-specific fluctuations in *Fv/Fm* and had a weaker overall effect. These *Fv/Fm* responses indicate a stronger physiological dependence on extracellular Ca^2+^ availability. Direct NMT measurements further showed that hypersaline stress rapidly induced Ca^2+^ influx and that Verapamil significantly inhibited this influx (Figure 2), supporting extracellular Ca^2+^ influx as a major contributor to the early Ca^2+^ response of *P. haitanensis* under hypersaline stress. Further Ca^2+^ supplementation experiments showed that, under Ca^2+^-free hypersaline treatment, Ca^2+^ supplementation within 2 h of stress restored *Fv/Fm* to a level similar to that under hypersaline treatment with Ca^2+^, whereas supplementation after 4 h failed to restore *Fv/Fm* (Figure 1D–G). This result suggests that, during the early stage of stress in the absence of exogenous Ca^2+^, intracellular Ca^2+^ release may partially contribute to maintaining physiological stability and stress-response capacity. However, as stress continues, prolonged Ca^2+^ limitation may aggravate physiological damage and reduce the recovery capacity of the thalli. Therefore, Ca^2+^ signaling plays a key role in the early salt stress response of *P. haitanensis*.

In higher plants, the SOS pathway is an important mechanism regulating Na^+^ efflux. Salt stress-induced Ca^2+^ signals can further regulate SOS1-mediated Na^+^ efflux through the SOS pathway [35,36,37]. In this study, hypersaline stress induced Ca^2+^ influx in *P. haitanensis*, while Na^+^ and K^+^ both shifted from influx under normal conditions to efflux under hypersaline stress (Figure 2B,C). After Verapamil was added to inhibit Ca^2+^ influx, both Na^+^ and K^+^ efflux were significantly suppressed (Figure 2D–F), suggesting a close association between extracellular Ca^2+^ influx and Na^+^/K^+^ flux regulation in *P. haitanensis* under hypersaline stress. In contrast, after the SOS1 inhibitor Amiloride was added, Na^+^ flux shifted from efflux to influx, while Ca^2+^ influx was enhanced and K^+^ flux showed no significant change (Figure 2D–F). Steinhorst et al. (2022) found that, in *Arabidopsis*, both the amplitude of the initial salt-induced Ca^2+^ signal and the propagation rate of the subsequent Ca^2+^ signal increased with increasing Na^+^ concentration, indicating that Ca^2+^ signaling can respond to changes in Na^+^ concentration in a dose-dependent manner [38]. Therefore, in this study, the inhibition of Na^+^ efflux accompanied by enhanced Ca^2+^ influx suggests that blocked Na^+^ efflux may aggravate cellular Na^+^ stress, thereby strengthening the Ca^2+^ signaling response and regulating subsequent ion homeostasis.

### 4.2. Identification and Expression Characteristics of Sos Pathway-Related Genes in P. haitanensis

In the SOS pathway, SOS1, SOS2, and SOS3 participate in Na^+^ efflux, protein kinase-mediated regulation, and Ca^2+^ signal perception, respectively, and are key components for maintaining ion homeostasis under salt stress in plants [5,37]. In this study, 5 *PhSOS1*, 3 *PhSOS2L*, and 6 *PhSOS3L* candidate genes were identified in the reference genome of *P. haitanensis* (Figure 3A). Among them, PhSOS1 and PhSOS3L proteins retained the core domains characteristic of plant SOS1 and SOS3 proteins, respectively. Notably, the number of EF-hand motifs varied among the 6 PhSOS3L proteins, and most members contained fewer EF-hand motifs than typical SOS3 proteins, which generally contain 4 EF-hand motifs [39]. Meanwhile, although all PhSOS2L proteins contained the serine/threonine protein kinase domain (S_TKc), the C-terminal CIPK_C regulatory domain typical of SOS2 proteins was not detected. These results indicate that *P. haitanensis* retains core SOS pathway-related components, but its Ca^2+^-sensing proteins and protein kinase modules exhibit distinct structural features, suggesting that the SOS-related regulatory module in *P. haitanensis* may differ from the classical SOS pathway in higher plants.

To investigate the salt stress response characteristics of SOS candidate genes in *P. haitanensis*, their expression patterns under hypersaline treatment were further examined. The results showed that, except for *PhSOS2L-1*, all candidate genes were significantly upregulated at approximately 10 min after salt stress treatment, and some genes showed a second upregulation at 30 min (Figure 3B–D and Figure S3). Previous studies have shown that SOS pathway-related genes in tomato [40], maize [41], wheat [42], rice [43], and other plants are usually significantly upregulated several hours or even longer after salt treatment. Notably, in this study, most SOS candidate genes in *P. haitanensis* were significantly induced at approximately 10 min after hypersaline treatment, indicating that these SOS candidate genes have a rapid response characteristic under hypersaline stress.

After Verapamil was further added, the four *PhSOS1* candidate genes showed a downregulated trend at all time points (Figure 3E and Figure S4), indicating that *PhSOS1* expression is regulated by extracellular Ca^2+^ influx. This is consistent with the role of SOS1 as a downstream effector of calcium signaling in the classical SOS pathway [44,45]. In contrast, after Amiloride was added, only *PhSOS1-3* among the *PhSOS1* genes was significantly inhibited, *PhSOS2L-1* was significantly upregulated, *PhSOS2L-2* was suppressed, and *PhSOS3L* genes generally showed an upregulated trend (Figure 3E–G and Figure S4). Studies have shown that Amiloride can inhibit Na^+^/H^+^ antiport activity by competing with Na^+^ for transporter binding sites [46]. Therefore, in this study, after Amiloride treatment, Na^+^ flux shifted from efflux to influx, accompanied by enhanced Ca^2+^ influx and upregulated expression of some *PhSOS1* and *PhSOS3L* genes. This may reflect a compensatory regulatory response after Na^+^ efflux was blocked. That is, if intracellular Na^+^ further accumulates due to impaired efflux, thalli may strengthen Ca^2+^ signaling and SOS-related gene responses to alleviate ion toxicity caused by Na^+^ accumulation. Meanwhile, the response differences among *PhSOS1* and *PhSOS2L* members suggest that functional differentiation or regulatory divergence may exist among SOS candidate genes in *P. haitanensis*.

Phosphorylation is a key process in signal transduction within the SOS pathway [47,48]. In *Arabidopsis*, the SOS2-SOS3 complex can relieve the auto-inhibited state of SOS1 by phosphorylating its C-terminal regulatory region, thereby activating SOS1-mediated Na^+^/H^+^ exchange activity. Mutations in the corresponding phosphorylation sites significantly weaken SOS1 function [37]. Similarly, mutation of key sites in wheat *TdSOS1* markedly affects its phosphorylation level and Na^+^ efflux function [49]. In this study, SOS candidate proteins in *P. haitanensis* underwent differential phosphorylation during salt stress. Among them, *PhSOS1-4* contained the largest number of differentially phosphorylated sites, with 11 sites identified. In addition, both the transcript level and protein abundance of *PhSOS1-4* increased significantly under salt stress (Figure 3E,H and Figure S5). These multilayered response characteristics, together with the conserved Na_H_Exchanger domain and the predicted plasma membrane localization, highlight PhSOS1-4 as a key candidate among the five identified PhSOS1 members potentially involved in Na^+^ efflux regulation in *P. haitanensis.* However, the Na^+^/H^+^ exchange activity of PhSOS1-4 has not been directly measured, and its predicted plasma membrane localization has not yet been experimentally validated. Therefore, the specific Na^+^/H^+^ antiporter function of PhSOS1-4 remains to be further experimentally confirmed.

### 4.3. Interaction Analysis and Functional Validation of Key Components of the Sos-like Pathway in P. haitanensis

In higher plants, the classical SOS signaling pathway generally follows a cascade activation model of SOS3-SOS2-SOS1 [50,51]. Whether *PhSOS1-4* is regulated through a classical SOS cascade is a key question for understanding the regulatory characteristics of the SOS pathway in *P. haitanensis*. Yeast two-hybrid screening showed that PhSOS1-4 stably interacted with PhSOS3L-5 (Figure 4A). Meanwhile, PhSOS1-4 also interacted with PhSOS2L-2 (Figure 4B), whereas no direct interaction between PhSOS2L-2 and PhSOS3L-5 was detected under the Y2H conditions used in this study (Figure 4C). This interaction pattern differs from the classical linear “SOS3-SOS2-SOS1” model in higher plants and suggests the possibility of a distinct SOS-related interaction mode in *P. haitanensis*. However, the absence of a detectable interaction between PhSOS2L-2 and PhSOS3L-5 in the Y2H assay does not rule out their possible interaction under native cellular conditions.

Studies have shown that Ca^2+^-sensing proteins do not act only indirectly through CIPK kinases, but can also directly participate in the regulation of membrane transport processes. For example, the *Arabidopsis* CaM-like protein AtCaM15 directly interacts with the C-terminal region of the tonoplast Na^+^/H^+^ antiporter AtNHX1, and this interaction is Ca^2+^- and pH-dependent [52]. Therefore, the Y2H-detected interaction between PhSOS3L-5 and PhSOS1-4 raises the possibility of a more direct mode of Ca^2+^ signal transduction. Combined with the rapid enhancement of Ca^2+^ influx under hypersaline stress and the rapid response of PhSOS1 genes, this interaction pattern may potentially help *P. haitanensis* transmit Ca^2+^ signals to the regulation of Na^+^ efflux during the early stage of stress. In addition, truncation validation further showed that the interaction between PhSOS1-4 and PhSOS3L-5 depended on the N-terminal non-domain region corresponding to amino acids 1–54 of PhSOS1-4 (Figure S6). Previous studies have shown that the N-terminal region of *Arabidopsis* SOS1 contains hydrophobic transmembrane domains, which provide an important structural basis for Na^+^/H^+^ antiporter activity [53]. This result further supports the involvement of the N-terminal region of PhSOS1-4 in its interaction with PhSOS3L-5 under the Y2H conditions used.

The observation that PhSOS2L-2 interacted with PhSOS1-4 but not with PhSOS3L-5 further indicates that PhSOS2L-2 in *P. haitanensis* may not fully follow the typical model in which SOS2 first forms a complex with SOS3 and then regulates SOS1. Together with the absence of the typical C-terminal CIPK_C regulatory domain in PhSOS2L proteins, PhSOS2L-2 may lack the structural basis required to form a classical CBL-CIPK complex with PhSOS3L-5. On the other hand, PhSOS2L-2 still interacted with PhSOS1-4, suggesting that it may act as a candidate protein kinase that directly interacts with PhSOS1-4 and potentially participates in its phosphorylation regulation. Similar “kinase-transporter” regulatory relationships have been reported in plants. For example, under low-K^+^ stress, *Arabidopsis* CIPK23 can be activated by CBL1/9 and can also directly phosphorylate and regulate the K^+^ transporter AKT1 [54].

Heterologous expression results showed that the biomass of *PhSOS1-4*- and *PhSOS3L-5*-transgenic *C. reinhardtii* was significantly higher than that of the wild type under high-salt stress, and both genes were significantly induced after salt stress (Figure 5G,I,J,L). These results indicate that heterologous expression of *PhSOS1-4* and *PhSOS3L-5* contributes to enhanced high-salt tolerance in *C. reinhardtii*. This is consistent with reports that overexpression of SOS1 improves salt tolerance in higher plants [55,56,57]. It also supports the possibility that *PhSOS3L-5*, as a Ca^2+^-sensing protein, plays a positive role in the salt stress response [58].

In contrast, the biomass of *PhSOS2L-2*-transgenic *C. reinhardtii* was significantly lower than that of the wild type under high-salt stress, showing a reduced salt tolerance phenotype (Figure 5H,K). This result is consistent with the lack of interaction between PhSOS2L-2 and PhSOS3L-5, suggesting that the functional output of PhSOS2L-2 is strongly dependent on the system background and is not fully equivalent to the regulatory mode of classical SOS2. In the classical SOS pathway, SOS2 functions as a serine/threonine protein kinase, and its activation usually depends on binding to a specific calcium sensor to relieve auto-inhibition [59]. In yeast, *Arabidopsis* SOS2 alone cannot activate downstream SOS1, and co-expression with SOS3 is required to restore the salt tolerance of the mutant [45]. Therefore, in the *C. reinhardtii* heterologous expression system, *PhSOS2L-2* may fail to establish an effective regulatory state due to the absence of specific upstream activators, interaction networks, or subcellular environments from *P. haitanensis*, and may even interfere with the host protein phosphorylation and ion homeostasis networks. Similarly, overexpression of *CdtCIPK21* from *Cynodon dactylon* in rice significantly reduces salt tolerance, accompanied by decreased ABA sensitivity and reduced antioxidant enzyme activity [60], indicating that the function of CIPK-type kinases in heterologous systems may be affected by the host background. Taken together, the heterologous expression results further support the possibility that the SOS-like regulatory mode involved in the salt stress response of *P. haitanensis* differs from the classical SOS regulatory model in terrestrial plants.

## 5. Conclusions

**Figure 6:**
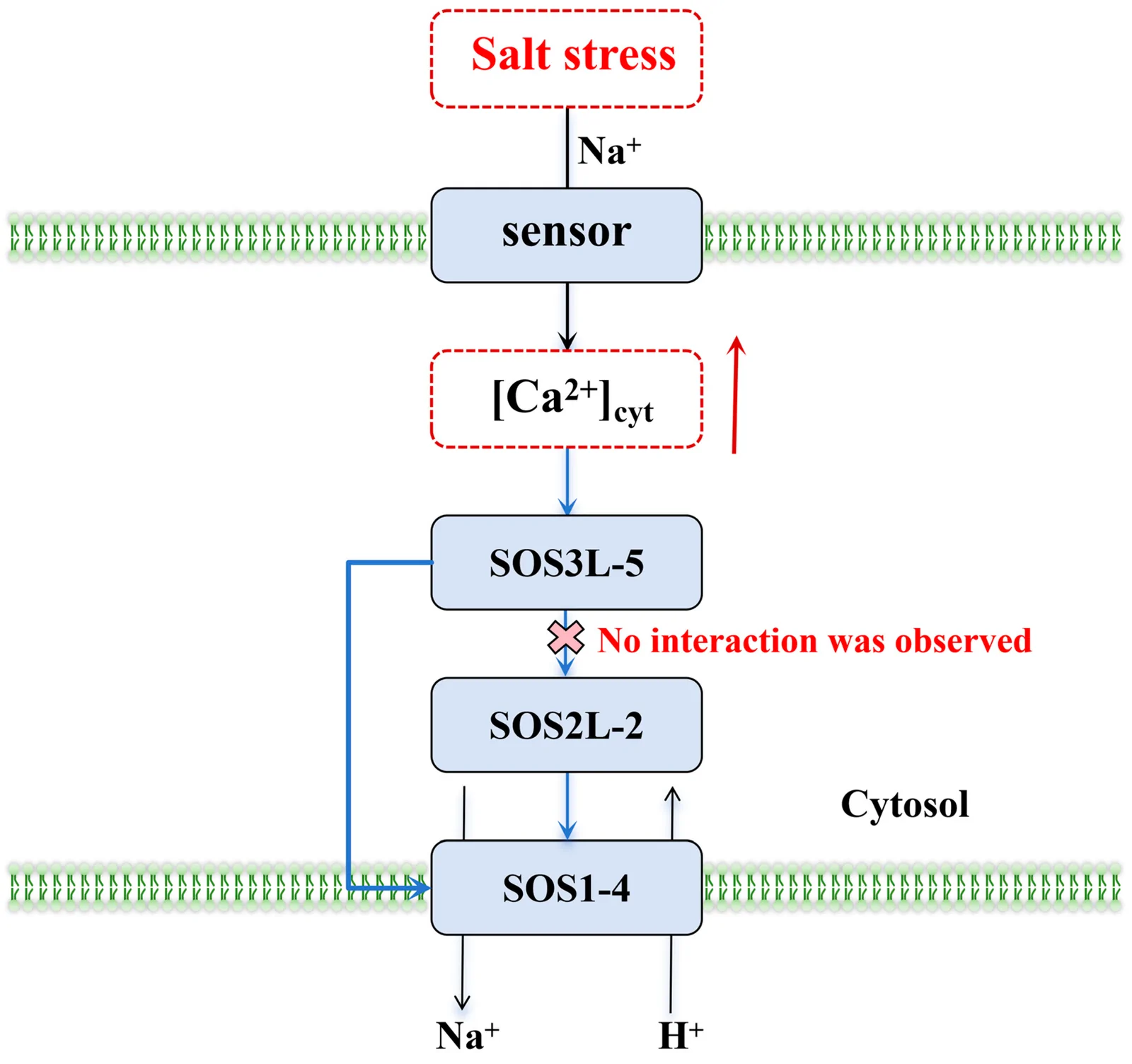
Hypothetical model of the SOS-like signaling pathway involved in the salt stress response of *P. haitanensis*. Salt stress induces Na^+^ stress signals and rapidly activates a cytosolic Ca^2+^ response mainly driven by extracellular Ca^2+^ influx. After sensing Ca^2+^ signals, PhSOS3L-5 may directly interact with PhSOS1-4 and potentially contribute to Na^+^ efflux regulation through this interaction. PhSOS2L-2 can also interact with PhSOS1-4, whereas no direct interaction was detected between PhSOS3L-5 and PhSOS2L-2.

Hypersaline stress rapidly induced a Ca^2+^ signaling response dominated by extracellular Ca^2+^ influx within 15 min in *P. haitanensis*, and this response was closely associated with Na^+^ efflux and concomitant changes in K^+^ flux. Based on the reference genome, a total of 14 SOS pathway-related candidate genes were identified, most of which were significantly upregulated at approximately 10 min after hypersaline treatment, showing a rapid response pattern. *PhSOS1-4* responded significantly at the transcriptional, protein abundance, and phosphorylation levels and was predicted to localize to the plasma membrane, highlighting it as a key candidate among the identified PhSOS1 members potentially involved in Na^+^ efflux regulation in *P. haitanensis*. Further analysis showed that PhSOS1-4 interacted with PhSOS3L-5 and PhSOS2L-2, respectively, whereas no direct interaction was detected between PhSOS3L-5 and PhSOS2L-2. Meanwhile, heterologous expression of both *PhSOS1-4* and *PhSOS3L-5* enhanced the hypersaline tolerance of *C. reinhardtii*. Taken together, these results support the hypothesis that *P. haitanensis* may possess an SOS-like regulatory mode that differs from the classical linear cascade model of the SOS pathway in higher plants. In this hypothetical pathway, after sensing Ca^2+^ signals, PhSOS3L-5 may rapidly and directly interact with PhSOS1-4 and potentially contribute to Na^+^ efflux regulation through this interaction, thereby contributing to faster signal transduction and ion homeostasis adjustment during the early stage of hypersaline stress in *P. haitanensis* (Figure 6).

## Data Availability

The data supporting the findings of this study are available from the authors upon request.

## Supplementary Materials

The following supporting information can be downloaded at https://www.mdpi.com/article/10.3390/plants15203188/s1: Figure S1. EF-hand motif distribution of PhSOS3L candidate proteins in *P. haitanensis*. (A–F) correspond to PhSOS3L-1 to PhSOS3L-6, respectively. Green boxes indicate EF-hand motifs, and the scale below each protein represents amino acid positions; Figure S2. Stage-specific detection of PhSOS1-2 in *P. haitanensis*. M indicates the DNA marker. P1–P4 represent four primer pairs targeting PhSOS1-2. Amplification bands were detected in the conchocelis but not in the thallus, indicating stage-specific detection of PhSOS1-2; Figure S3. Expression patterns of additional PhSOS candidate genes in *P. haitanensis* under hypersaline stress. Relative expression levels of PhSOS1-1, PhSOS1-3, PhSOS1-5, PhSOS2L-2, PhSOS3L-1, PhSOS3L-2, PhSOS3L-4, and PhSOS3L-6 were determined after hypersaline treatment for 0, 5, 10, 15, 30, and 60 min. Different letters indicate significant differences among different treatment times (*p* < 0.05); Figure S4. Expression patterns of additional PhSOS candidate genes in *P. haitanensis* under hypersaline stress after inhibitor treatments. Relative expression levels of PhSOS1-1, PhSOS1-3, PhSOS1-5, PhSOS2L-2, PhSOS3L-1, PhSOS3L-2, PhSOS3L-4, and PhSOS3L-6 were determined after 0, 5, 10, 15, 30, and 60 min of treatment. S110 indicates 110‰ hypersaline treatment; S110 + Verapamil indicates hypersaline treatment with the Ca2+ channel inhibitor Verapamil; S110 + Amiloride indicates hypersaline treatment with the SOS1 inhibitor Amiloride. * and ** indicate significant and highly significant differences between treatments, respectively (*p* < 0.05 and *p* < 0.01); Figure S5. Protein abundance of PhSOS1-4 in *P. haitanensis* under hypersaline stress. Western blot analysis showed the abundance of PhSOS1-4 after hypersaline treatment for 0, 5, 10, 15, 30, and 60 min. CK represents the untreated control, and RbcL was used as the loading control. Relative expression was normalized to RbcL, with CK set to 1; Figure S6. Interaction validation between PhSOS3L-5 and different truncated fragments of PhSOS1-4. (A–C) One-to-one yeast two-hybrid validation was performed between pPR3-N-PhSOS3L-5 and different truncated fragments of PhSOS1-4 used as bait proteins. (A) interaction validation between pBT3-STE-PhSOS1-41–498aa and pPR3-N-PhSOS3L-5. (B) interaction validation between pBT3-STE-PhSOS1-455–1593aa and pPR3-N-PhSOS3L-5. (C) interaction validation between pDHB1-PhSOS1-41–54aa and pPR3-N-PhSOS3L-5. In each panel, the left plate shows growth on DDO medium for detecting plasmid co-transformation, and the right plate shows growth and color development on QDO/X-Gal selective medium supplemented with 3-AT for detecting protein interaction; Table S1. Primer sequences used for qRT-PCR and PCR; Table S2. Subcellular localization prediction of SOS candidate proteins in *P. haitanensis* using WoLF PSORT; Table S3. Information of valid clones obtained from yeast two-hybrid screening using PhSOS1-4 as bait.
