# Supplementary material for: Characterization of Ca2+-Dependent SOS-like Pathway Components Involved in the Salt Stress Response of an Intertidal Macroalga, Pyropia haitanensis
Source: Plants (Basel). 2026 Oct 17;15(20):3188. doi: 10.3390/plants15203188 (PMC13708748; doi:10.3390/plants15203188)
Supplement: Supplementary file 1 [file plants-15-03188-s001.zip › Supplementary material.pdf]

**Supplementary material**

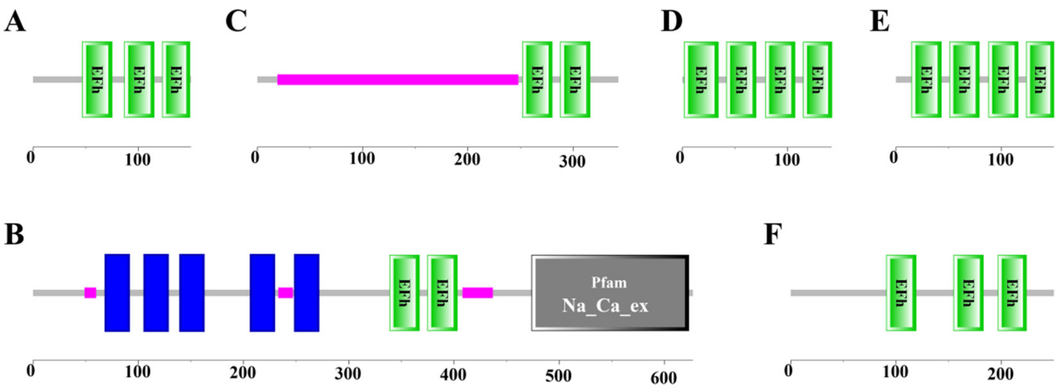


**Figure S1. EF-hand motif distribution of PhSOS3L candidate proteins in *P. haitanensis*.** (**A-F**) correspond to PhSOS3L-1 to PhSOS3L-6, respectively. Green boxes indicate EF-hand motifs, and the scale below each protein represents amino acid positions.

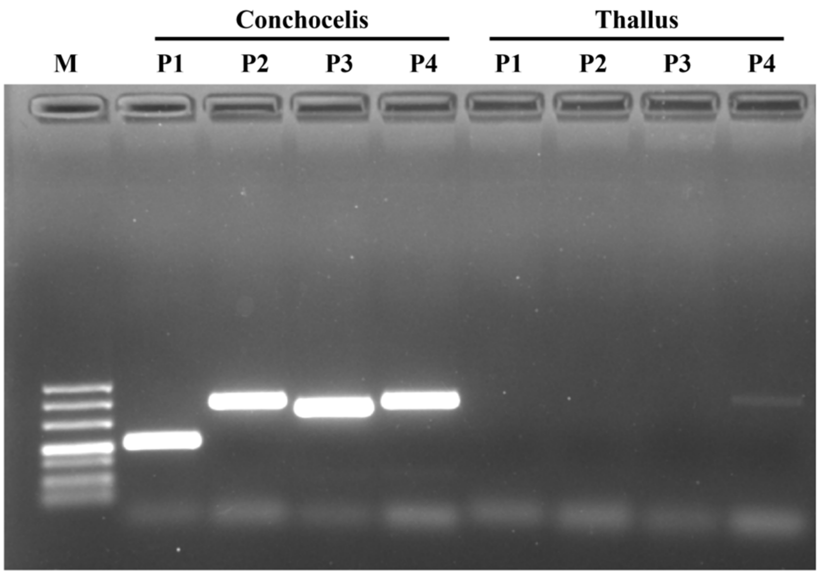


**Figure S2. Stage-specific detection of *PhSOS1-2* in *P. haitanensis*.** M indicates the DNA marker. P1-P4 represent four primer pairs targeting *PhSOS1-2*. Amplification bands were detected in the conchocelis but not in the thallus, indicating stage-specific

detection of *PhSOS1-2*.

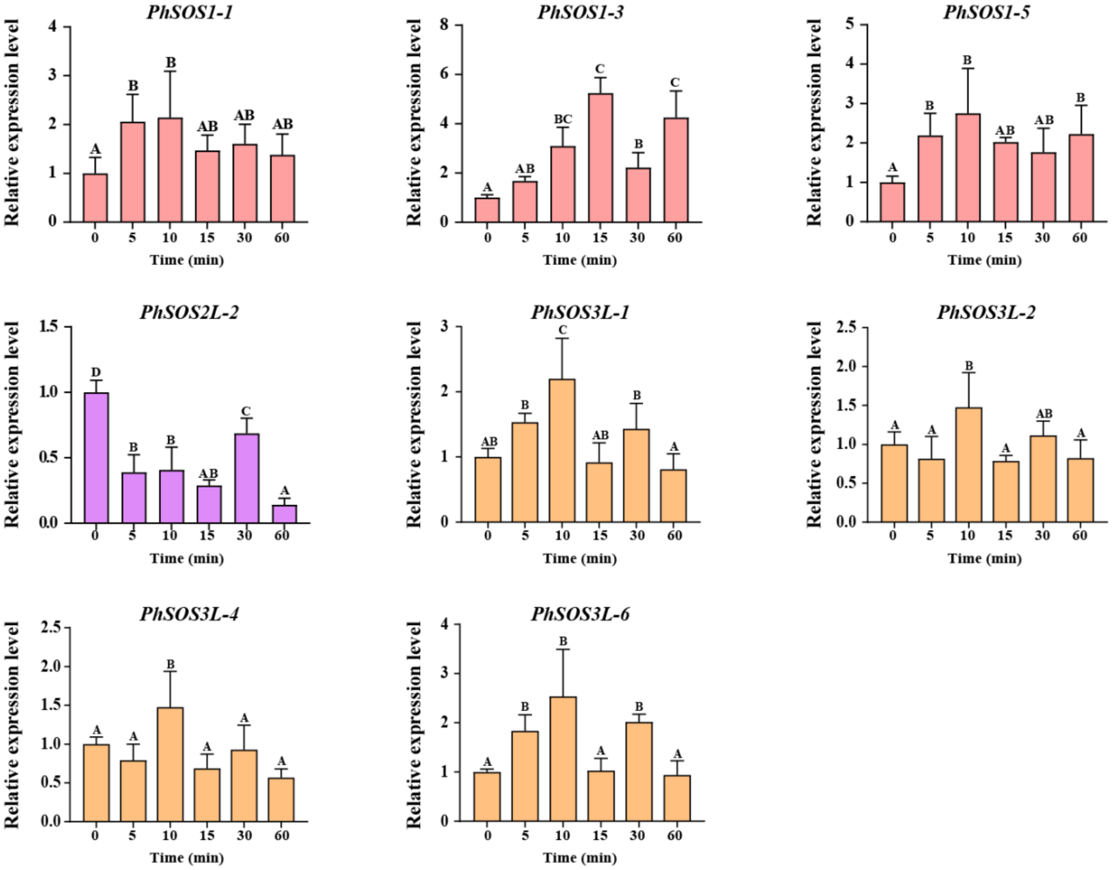


**Figure S3. Expression patterns of additional *PhSOS* candidate genes in *P. haitanensis* under hypersaline stress.** Relative expression levels of *PhSOS1-1*, *PhSOS1-3*, *PhSOS1-5*, *PhSOS2L-2*, *PhSOS3L-1*, *PhSOS3L-2*, *PhSOS3L-4*, and *PhSOS3L-6* were determined after hypersaline treatment for 0, 5, 10, 15, 30, and 60 min. Different letters indicate significant differences among different treatment times ($P < 0.05$).

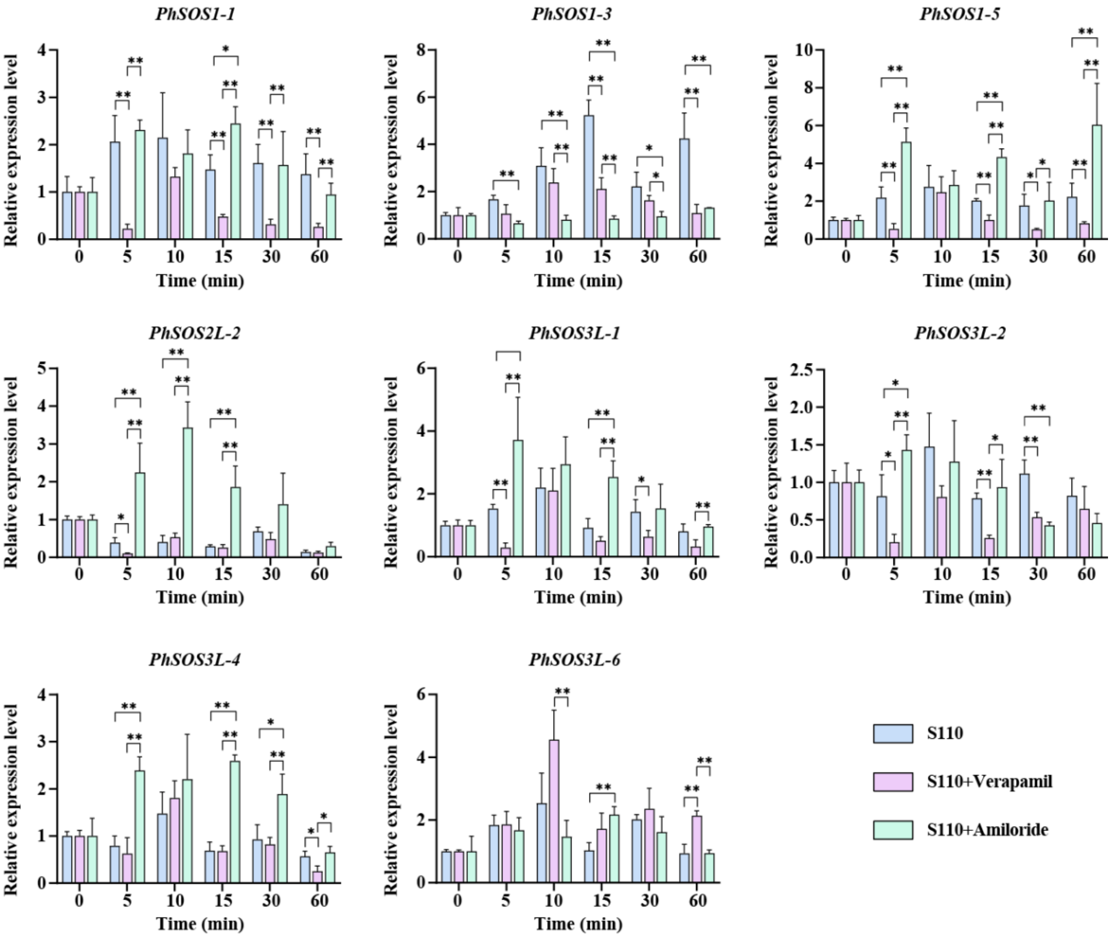


**Figure S4. Expression patterns of additional *PhSOS* candidate genes in *P. haitanensis* under hypersaline stress after inhibitor treatments.** Relative expression levels of *PhSOS1-1*, *PhSOS1-3*, *PhSOS1-5*, *PhSOS2L-2*, *PhSOS3L-1*, *PhSOS3L-2*, *PhSOS3L-4*, and *PhSOS3L-6* were determined after 0, 5, 10, 15, 30, and 60 min of treatment. S110 indicates 110‰ hypersaline treatment; S110 + Verapamil indicates hypersaline treatment with the $Ca^{2+}$ channel inhibitor Verapamil; S110 + Amiloride indicates hypersaline treatment with the SOS1 inhibitor Amiloride. * and ** indicate significant and highly significant differences between treatments, respectively ($P < 0.05$ and $P < 0.01$).

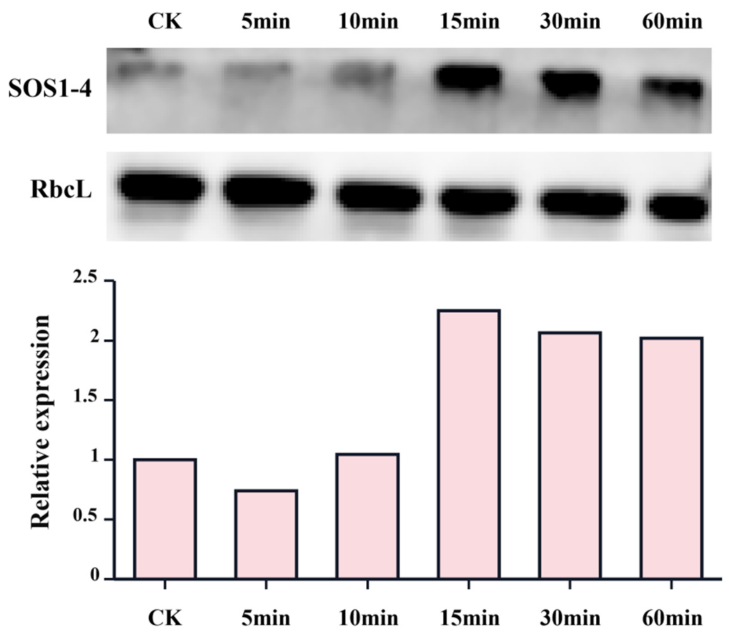


**Figure S5. Protein abundance of PhSOS1-4 in *P. haitanensis* under hypersaline stress.** Western blot analysis showed the abundance of PhSOS1-4 after hypersaline treatment for 0, 5, 10, 15, 30, and 60 min. CK represents the untreated control, and RbcL was used as the loading control. Relative expression was normalized to RbcL, with CK set to 1.

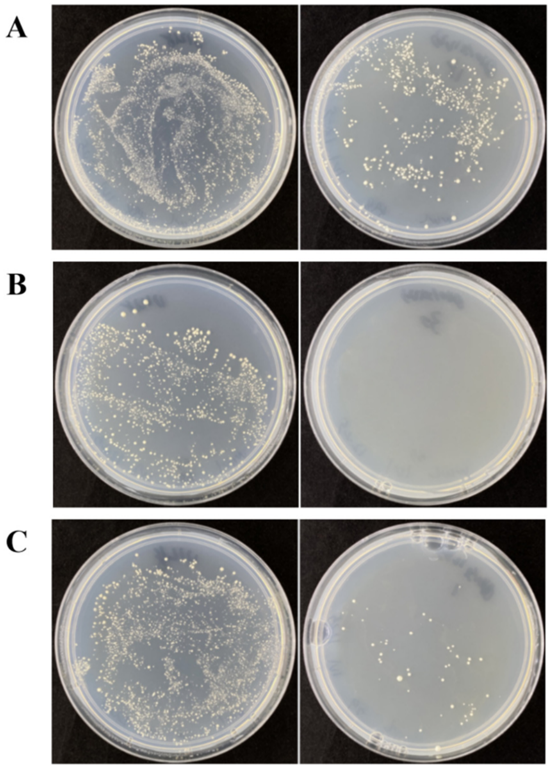

**Figure S6. Interaction validation between PhSOS3L-5 and different truncated fragments of PhSOS1-4. (A-C)** One-to-one yeast two-hybrid validation was performed between pPR3-N-PhSOS3L-5 and different truncated fragments of PhSOS1-4 used as bait proteins. **(A)** interaction validation between pBT3-STE-PhSOS1-41–498aa and pPR3-N-PhSOS3L-5. **(B)** interaction validation between pBT3-STE-PhSOS1-455–1593aa and pPR3-N-PhSOS3L-5. **(C)** interaction validation between pDHB1-PhSOS1-41–54aa and pPR3-N-PhSOS3L-5. In each panel, the left plate shows growth on DDO medium for detecting plasmid co-transformation, and the right plate shows growth and color development on QDO/X-Gal selective medium supplemented with 3-AT for detecting protein interaction.

**Table S1. Primer sequences used for qRT-PCR and PCR.**

**Table S2. Subcellular localization prediction of SOS candidate proteins in *P. haitanensis* using WoLF PSORT.**

**Table S3. Information of valid clones obtained from yeast two-hybrid screening using PhSOS1-4 as bait.**
